# Training a machine learning classifier to identify ADHD based on real-world clinical data from medical records

**DOI:** 10.1038/s41598-022-17126-x

**Published:** 2022-07-28

**Authors:** Pavol Mikolas, Amirali Vahid, Fabio Bernardoni, Mathilde Süß, Julia Martini, Christian Beste, Annet Bluschke

**Affiliations:** 1 Department of Child and Adolescent Psychiatry and Psychotherapy, Carl Gustav Carus University Hospital, Technische Universität Dresden, Fetcherstr. 74, 01307 Dresden, Germany; 2 Department of Psychiatry and Psychotherapy, Carl Gustav Carus, University Hospital, Technische Universität Dresden, Fetscherstraße 74, 01307 Dresden, Germany; 3 Translational Developmental Neuroscience Section, Division of Psychological and Social Medicine and Developmental Neuroscience, Faculty of Medicine, Technische Universität Dresden, Dresden, Germany

**Keywords:** ADHD, Diagnosis

## Abstract

The diagnostic process of attention deficit hyperactivity disorder (ADHD) is complex and relies on criteria sensitive to subjective biases. This may cause significant delays in appropriate treatment initiation. An automated analysis relying on subjective and objective measures might not only simplify the diagnostic process and reduce the time to diagnosis, but also improve reproducibility. While recent machine learning studies have succeeded at distinguishing ADHD from healthy controls, the clinical process requires differentiating among other or multiple psychiatric conditions. We trained a linear support vector machine (SVM) classifier to detect participants with ADHD in a population showing a broad spectrum of psychiatric conditions using anonymized data from clinical records (N = 299 participants). We differentiated children and adolescents with ADHD from those not having the condition with an accuracy of 66.1%. SVM using single features showed slight differences between features and overlapping standard deviations of the achieved accuracies. An automated feature selection achieved the best performance using a combination 19 features. Real-world clinical data from medical records can be used to automatically identify individuals with ADHD among help-seeking individuals using machine learning. The relevant diagnostic information can be reduced using an automated feature selection without loss of performance. A broad combination of symptoms across different domains, rather than specific domains, seems to indicate an ADHD diagnosis.

## Introduction

The diagnostic process in the case of suspected attention deficit hyperactivity disorder (ADHD) commonly entails collecting a substantial amount of data and is thus complex, time-consuming and costly. A substantial amount of data, however, is necessary to distinguish whether ADHD underlies the particular pattern of observed symptoms as opposed to norm variants of behavior, possible differential diagnoses, or comorbidities occurring in addition to ADHD^1,2^. Overall, this extensive diagnostic process relies on criteria highly sensitive to subjective biases (for discussion see Faraone et al.^3^) and may result in delays in treatment initiation. This is particularly unfortunate given that effective treatments for ADHD are readily available. Thus, it should be paramount to streamline, shorten and specify the diagnostic process. To achieve this, it is necessary to identify the most relevant aspects of data that predict the diagnostic outcome. This may be possible via the application of machine learning techniques.

In recent years, machine learning has made remarkable progress, from its application in detecting between-group differences to making predictions on the individual level^4^. Concerning ADHD, previous studies based on clinical and/or neuroimaging data have performed automated classifications to distinguish between ADHD and typically developing individuals with classification accuracies ranging from 62 to 89.5%^5–9^. Unfortunately, this dichotomous distinction between the labels of "typically developing" and "ADHD" does not reflect the question typically asked in the clinical setting. Even though this question is clinically much more relevant, to our knowledge no study so far has attempted to apply machine learning in order to predict whether the diagnostic outcome will be "ADHD" or "something else" (i.e., a norm variant of behaviour or another psychiatric diagnosis) in a broad spectrum of clinical conditions within a help-seeking population.

Since neuroimaging or genetic data are not (yet) part of the routine diagnostic process for ADHD due to limitations in cross-sample reliability/validity as well as in sensitivity and specificity^10^ and may result in lower classification accuracy than clinical measures^5,6^, it is currently still necessary to focus on readily available behavioural/clinical data including demographic information, subjective symptom ratings, and objective neuropsychological data.

Demographic data like male gender, severe early onset and familial predispositions^11^ are associated with a higher risk for ADHD. Self-report symptom rating scales, are less reliable than informant ratings^12^ with studies further reporting low to medium correlations between parent and teacher ratings^13^. To account for these differences, it has been suggested to use the degree of consistency between them as an indicator of ADHD symptom severity^14^.

Neuropsychological tests are a further important component of the data collected during the diagnostic process. Lower overall IQ^15^, as well as difficulties in working memory^1^ and processing speed^16,17^, have been proposed to distinguish between individuals with ADHD and typically developing controls. Verbal comprehension and logical reasoning, in turn, are not systematically reduced in children with ADHD^18^. Overall, reductions in general or subscale-specific IQ are not specific to ADHD. Instead, the label "ADHD" explicitly points to difficulties in attentional processes (e.g. Günther et al.^19^). Evidence for impairments in terms of accuracy and reaction time variability^20^ in tests pertaining to inhibition^21^ seems to be particularly strong. Similarly, this is the case regarding the intensity domain of attention^22^. Specifically, this concerns omission errors occurring in tests of sustained attention/tonic alertness^23^. Evidence is rather mixed concerning the selectivity domain of attention^21,22^.

In this proof-of-concept study, we attempted to train a machine learning model to predict the diagnostic outcome of "ADHD" in a help-seeking clinical sample. To our knowledge, this is the first study that attempted to train a machine learning classifier on anonymized real-world clinical data and to distinguish children/adolescents with an ADHD diagnosis from those with none or other diagnoses. In addition to well-established neuropsychological measures and individual symptom ratings, we included features capturing the degree of consistency between parents' and teachers' ratings. In order to test possible implications for shortening the diagnostic process, we assessed the predictive information of every single feature. Moreover, we attempted to reduce the necessary diagnostic information using a data driven, automated feature selection.

## Methods

### Participants

The standardized diagnostic process included several consultations with the child and caregivers together and individually. Parents and (nursery) school teachers completed general and ADHD-specific rating scales. Further, general intelligence and attention were assessed via standardized testing batteries. In addition, somatic conditions which may contribute to any existing attention problems were excluded (e.g., laboratory measures, ophthalmological and ENT evaluations, EEG). The final diagnostic decision was given strictly based on ICD-10 clinical criteria assessed by a senior specialist in child and adolescent psychiatry or psychology.

This was a study based exclusively on data from a clinical records. We extracted the data of help-seeking individuals who were referred to our secondary care outpatients unit with a suspected ADHD diagnosis, or in whom an ADHD diagnosis was the suspected diagnosis after the initial consultation. The group labeled "ADHD" included patients who had received one of the following diagnoses: attention deficit hyperactivity disorder (F90.0), hyperkinetic conduct disorder (F90.1), or attention deficit disorder without hyperactivity (F98.80). Importantly, not all psychiatric comorbidities constituted an exclusion criterion for the ADHD group (see below). The "non-ADHD" group contained patients who did not fulfill diagnostic criteria for ADHD. Socio-demographic and clinical characteristics of the sample (N = 299) are presented in Table 1. Individuals who were classified in the group ADHD were significant more often male (chi^2^ = 6.871, *p* = .009) and younger (*t* = 2.038, *df* = 290, *p* = .043).

**Table 1:** Socio-demographic characteristics of the sample.

Variables	ADHD	Non-ADHD	Test
N (N_total_ = 299)	153 (52.4)	139 (47.6)	
Sex male (%)	132 (86.3)	103 (74.1)	χ^2^(1) = 6.871, *p* = .009
Age	10.0 (2.4)	10.5 (2.5)	*t* = 2.038, *df* = 290, *p* = .043
Total IQ (SD)	96.8 (13.0)	96.3 (11.9)	*t* = − .353, *df* = 290, *p* = .724
**Diagnoses N (%)**			
***ADHD***			
1. Predominantly hyperactive-impulsive type	98 (64.0)	n/a	
2. Predominantly inattentive type	42 (27.5)	n/a	
3. Comorbidwith conduct disorder	13 (8.5)	n/a	
Adjustment disorders	12 (7.8)	48 (34.5)	
Affective disorders	1 (0.6)	3 (2.2)	
Autism spectrum disorders	0 (0)	0 (0)	
Conduct disorders	n/a	14 (1)	
Disorders of social functioning with onset specific to childhood and adolescence	4 (2.6)	6 (4.3)	
Eating disorders	1 (0.6)	1 (0.7)	
Emotional disorders with onset specific to childhood	14 (9.2)	16 (11.5)	
Intellectual disabilities	3 (1.9)	0 (0)	
Mental and behavioral disorders due to substance use	0 (0)	7 (5.0)	
Mixed disorders of conduct and emotions	0 (0)	5 (3.6)	
Specific developmental disorder of motor function	10 (6.5)	3 (2.2)	
Specific developmental disorders of scholastic skills	16 (10.5)	6 (4.3)	
Tic disorders	12 (7.8)	18 (12.9)	
Other	6 (3.9)	6 (4.3)	
No diagnosis	n/a	50 (36.0)	

Data sets were included in the study if ADHD had been suspected at the beginning of the diagnostic process, patients were younger than 18 years at the beginning of the diagnostic procedure, and if at least 2 out of 3 attention tests scores from the TAP diagnostic battery (for details, see below) were available^24^. Data sets were excluded if neurological or genetic disorders, endocrine disorders (incl. not corrected hypo- or hyperthyroidism), or other severe documented medical comorbidities on Axis IV had been identified.

### Data collection

We extracted data from medical records of the Department of Child and Adolescent Psychiatry and Psychotherapy at the Medical Faculty of the Technical University Dresden from 2015 to 2020. As we used anonymized data from a clinical register, in alignment with the Saxony Hospital Act §34 Section 1, the informed consent was waived by the Ethics Committee of the Medical Faculty of the Technische Universität Dresden, Germany (No: EK31012016), who also approved the study. The study was performed in accordance with the Declaration of Helsinki. Briefly, we extracted the following 92 features from the clinical records (for a detailed summary, see Supplementary table 1):

I. Demographic variables (age and gender);
II. Symptom ratings (Conners-3 parent/teacher ratings^25^; parent version of the Child Behavior Checklist (CBCL) and its school equivalent, the Teacher's report form (TRF)^26^; Strengths and Difficulties Questionnaire parents (SDQ-P) and teacher (SDQ-T) versions^27^). To account for age and gender differences amongst patients, we used normed T-values as features in all cases. Additionally, we computed a set of 'consistency indices' describing the consistency between parent and teacher ADHD specific Conners-3 ratings (for details see Supplementary note 1).
III. Neuropsychological measures (three subtests from the TAP, a commonly used German computer-based assessment of attention in children and adolescents^24^ was used to assess inhibition (GoNogo subtest), divided attention (Divided Attention subtest) and Alertness (Alertness subtest). The Wechsler Intelligence Scale for Children IV or V^28,29^ was used to measure general intelligence. To generate compatibility between versions IV and V, we used the average of the visual-spatial index and the fluid reasoning index as 'perceptual reasoning' in participants who completed the WISC V. For the attention measures, we used the T-values as features. For the intelligence measures, we used the standardized IQ scores as features.

### Machine learning classification

Prior to classification, we discarded all features with > 20% of missing values (N = 62 features), as well as all participants with > 20% of missing features (N = 150 participants, 49%, (i.e. n = 70 participants) from the ADHD group). We determined the 20% cutoff as a compromise solution to preserve a diverse set of features without too strongly negatively impacting the accuracy due to too many missing values^30^. The final dataset was comprised of 292 participants and 30 features (Table 1). As the support vector machine (SVM) classifier cannot handle missing values, some imputations were necessary in order to retain the most participants and features. We imputed the sample mean (continuous variables) or mode (discrete variables). As an alternative approach to data imputation, we performed a supplementary analysis after discarding all subjects containing missing data on a dataset of N = 248 (53.2% ADHD). Finally, to eliminate the effect of a different range of features on classification performance, all features were normalized into a z-score.

We used a linear SVM classifier to classify the participants into ADHD and *non*-ADHD groups in three ways. First, to assess the SVM classifier's performance on the whole dataset, we used the complete set of 30 features for training and testing. Second, we assessed the importance of single features for the classification by performing the classification using each *one single* feature at a time (i.e., we repeated the above-mentioned training and testing phase, including the *k*-fold crossvalidation described below using a single feature at a time, obtaining 30 single-feature classifiers). We chose this procedure rather than reporting the SVM weights, as those cannot be interpreted regarding the importance of single features^31^. Finally, to try and optimize the algorithm's performance, we aimed to eliminate irrelevant features in a data-driven way. Similar to our previous work^32^, we used the sequential floating forward selection (SFFS)^33,34^ implemented in MATLAB 2017a (Mathworks Inc.) for this purpose. In an SFFS feature selection, two separate algorithms are combined. The sequential forward selection (SFS) starts from an empty set of features and sequentially adds features that result in the highest classifier accuracy when combined with the already selected features. Sequential backward selection (SBS) works in the opposite direction by removing the feature, leading to higher accuracy. In SFFS, each feature selection step comprises SFS and SBS^32^. After adding each feature, we performed an SVM classification using the selected set of features. We performed the train and test procedures using a standard *k*-fold crossvalidation method (*k* = 10)^4,35,36^ (for details see Supplementary note 2 and Supplementary figure 1). We calculated the classification accuracy (i.e. the number of correct predictions divided by the number of all predictions made) as the average accuracy on all folds and reported the standard deviation of the achieved accuracies.

Since this was a population-based study, the ADHD and *non*-ADHD participants were not matched by age and gender. To check that the classification was based on ADHD-specific traits rather than predominantly demographic variables (age, sex), we compared correctly vs. incorrectly predicted participants using a *t* test and a chi-square test, where applicable. A significant difference in some demographic variables (e.g., age) would indicate that the classifier would have a limited validity/range of applicability. To further assess the contribution of demographic variables to the classification, we also performed a secondary analysis repeating the primary SVM classification using all the features listed in Table 2 except for age and sex.

**Table 2:** Ranking of features according to the classification accuracy when used as single feature in an SVM model.

Ranking	Accuracy	Feature	Note
1	0.576	Gender	Male/ female
2	0.575	Go/NoGo_standard deviation	Go/Nogo: standard deviation (T value)
3	0.572	TAP_Alertness_Tonic_reaction time_standard deviation	Tonic alertness reaction time (without warning signal): standard deviation (T value)
4	0.551	Go/NoGo_commission errors	Go/Nogo: false alarms
5	0.545	Conners_peer relations_m	Item from Conners-3 parent ratings
6	0.545	Processing speed	Processing speed based on WISC IV or V (in children aged < 6 WPPSI)
7	0.538	Age	Age (years)
8	0.531	Go/NoGo_ommission errors	Go/Nogo: omission errors
9	0.530	TAP_Alertness_Phasic_reaction time_standard deviation	Phasic alertness reaction time (without warning signal): standard deviation (T value)
10	0.527	Conners_inattention_m	Item from Conners-3 parent ratings
11	0.524	Conners_hyperactivity/impulsivity_t	Item from Conners-3 teacher ratings
12	0.524	TAP_Alertness_tonic_reaction time_reaction time	Tonic alertness reaction time (without warning signal): mean reaction time (T value)
13	0.524	Conners_aggression_t	Item from Conners-3 teacher ratings
14	0.524	Go/NoGo_reaction time	Go/Nogo: mean reaction time (T value)
15	0.524	Conners_negative impression_t	Item from Conners-3 teacher ratings
16	0.524	Conners_executive functions_m	Item from Conners-3 parent ratings
17	0.524	Conners_learning problems_m	Item from Conners-3 parent ratings
18	0.524	TAP_Alertness_Phasic_reaction time	Phasic alertness reaction time (with warning signal): mean reaction time (T value)
19	0.524	Conners_cognitive problems_t	Item from Conners-3 teacher ratings
20	0.524	Conners_negative impression_m	Item from Conners-3 parent ratings
21	0.524	Conners_inattention_r	Consistency index – parent vs. teacher ratings
22	0.523	WISC_General IQ	Total IQ based on based on WISC IV or V (in children aged < 6 WPPSI)
23	0.523	Conners_positive impression_m	Item from Conners-3 parent ratings
24	0.520	Conners_aggression_m	Item from Conners-3 parent ratings
25	0.518	Conners_inattention_t	Item from Conners-3 teacher ratings
26	0.517	Verbal comprehension	Verbal comprehension based on WISC IV or V (in children aged < 6 WPPSI)
27	0.514	Conners_hyperactivity/impulsivity_m	Item from Conners-3 parent ratings
28	0.500	Perceptual reasoning	Perceptual reasoning based on WISC IV or V (in children aged < 6 WPPSI)
29	0.500	Working memory	Working Memory based on WISC IV or V (in children aged < 6 WPPSI)
30	0.469	Conners_peer relations_t	Item from Conners-3 teacher ratings

## Results

### SVM classification

The classification using the complete set of 30 features yielded an average accuracy of 66.1% (obtained from the true label) (SD = 8%, sensitivity = 66.9%, specificity = 65.4%, AUC = 0.66). The classifier falsely identified 18.2% of ADHD patients as not having ADHD (type 2 error). Conversely, 15.8% of patients without ADHD were falsely identified as having the condition (type 1 error). The permutation test showed that the accuracy is higher than randomly assigned labels (*p* value = .001). The correctly and incorrectly classified participants did not significantly differ in age (*t* = − .733, *df* = 290, *p* = .464), gender (χ^2^(1) = .171, *p* = .679) and total IQ (*t* = 1.173, *df* = 290, *p* = .242).

**Figure 1:**
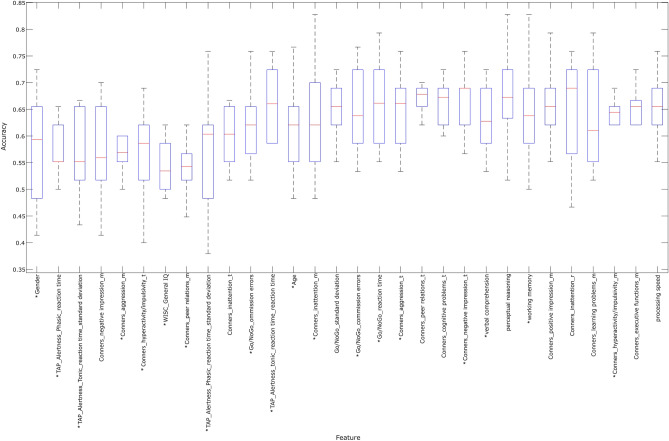
A box-plot diagram of the prediction accuracies achieved using only a single feature at a time. We chose this approach to evaluate, if some features might be more predictive than others. The bars indicate the standard deviation of the classification accuracies achieved in single runs of the tenfold crossvalidation method. The differences were not significant, as the standard deviations were high, and they overlapped, therefore we did not perform any further significance test. The * sign indicates those features, which, when combined, achieve the highest accuracy in a separate analysis (see Supplementary table 2).

We ranked the features according to the achieved classification accuracy when exclusively one feature was used for testing and training (Table 2). For a graphical interpretation including standard deviations, see Fig. 1.

The automated feature-selection procedure achieved a maximum classification accuracy of 68.1% using a set of 19 features (Supplementary table 2).

### Secondary classification without demographic features

In order to determine the predictive value of non-demographic features, we excluded the demographic features (age and sex) from training and classification in a secondary analysis. The model achieved an accuracy of 65.1% (sensitivity = 64.7%, specificity = 65.4%, AUC = 66.3%). A permutation test revealed this performance was significantly above chance (*p* = .001).

### Secondary classification without missing data

In order to relatively estimate the influence of missing data on the classification performance, we retrained the classifier using the automatically selected set of the 19 best predictive features (Supplementary table 2) only on subjects without any missing data. The SVM achieved an accuracy of 68.8% (SD = 8.5%, sensitivity = 63.3%, specificity = 73.9%, AUC = 69.6%).

## Discussion

In this machine learning study, we differentiated help-seeking children and adolescents with ADHD from those not having the condition with an accuracy of 66.1% using real-world clinical data from hospital records. Excluding demographic features (age and gender) resulted in a comparable accuracy. An automated feature selection achieved the best performance using a combination of 19 most predictive features across attention and intelligence domains and symptom ratings. The accuracy might be further increased using datasets without missing data. The consistency index of parent and teacher ratings did not outperform conventional features. Our study suggests that ADHD can be identified using data from clinical records even in a mixed, help-seeking population of children and adolescents.

Machine learning studies require large amounts of data^31^ which may be challenging to collect by recruiting participants for a specific study but are readily available in clinical databases. Moreover, the results from experimental studies might not generalize to a clinical setting, where clinicians are commonly confronted with multiple/concurrent disorders and/or various potential differential diagnoses. Thus, we showed that SVM in combination with real-world, comprehensive clinical data could yield an above-chance classification accuracy and detect individuals with ADHD among those having none or different condition(s).

To our knowledge, the highest achieved accuracy in studies of ADHD patients and healthy individuals were 89.5%^8^. Although we used more features than this study, the resulting accuracy was lower. This might be because many help-seeking individuals in our sample received other diagnoses associated with symptoms that may mimic ADHD (such as attention deficits in depression, increased activity in tic disorders, etc.). Thus, the two groups (ADHD vs. "something else") are not as clearly differentiated from each other as it would be the case when distinguishing between individuals with confirmed ADHD diagnoses and those not showing any symptoms at all. Previous studies aiming at distinguishing more than one disorder from typically developing controls reported lower classification accuracies than studies aiming at classifying typically developing individuals and patients with one condition^37^.

Age and gender were shown to be useful for diagnostic and prognostic tools based on machine learning in previous studies^38,39^. This was also the case in the current study. In this study, instead of identifying physiological patterns typical of ADHD, we aimed to train a classifier to identify ADHD based on data available from medical records. As typical age and gender distributions of ADHD may naturally be reflected in this data structure, which may constitute a sampling bias, conducting a second analysis without these features was essential. This analysis without age and gender still revealed a significant classification accuracy, demonstrating that the neuropsychological features and ADHD-specific ratings on their own are sufficient to identify ADHD in a mixed patient sample.

Previous studies have opted not to include clinical ratings in the analysis to avoid possible subjective biases^8^. We addressed this issue by using the consistency index above, which did not outperform conventional ADHD-specific features like parent/teacher-rated symptoms. The automatic feature selection also only emphasized a rather unspecific symptoms like peer relations, aggression, and teacher negative impression bias. These results suggest that clinical ratings capturing broader ADHD-related behavioral irregularities (i.e., not simply pertaining to ADHD core symptoms) as reported by different sources using the Conners-3 questionnaire are informative when aiming to identify ADHD amongst a help-seeking clinical population. This may reflect the notion that the rather qualitative "clinical impression" of ADHD plays a significant role in the diagnostic process^40^. Similarly, this may also be interpreted as showing that a rather broad functional impairment associated with ADHD symptoms (in regards to social interactions, for example) is indicative of diagnostic classification in the clinical setting. This issue could be examined further by including clinician rating scales or those capturing the degree of functional impairment^41^.

Among the neuropsychological measures, the total IQ score did not rank among the most predictive features. Previous machine learning studies suggesting IQ to be a predictive feature^5,6^ included IQ scores as part of an overall "phenotypic" feature that also contained aspects like age and gender, making a specific interpretation impossible. In addition, these studies only focused on the distinction between individuals with ADHD and typically developing controls, thus reducing the validity of the results for clinical practice. It is the goal to distinguish ADHD from disorders or norm variants of behavior mimicking ADHD symptoms. Interestingly, the processing speed subscale ranked highest of all IQ-related features in the single feature classification. This may reflect the previously reported relevance of this aspect of neuropsychological processing^16^ when comparing individuals with ADHD and healthy controls. Within the automatic feature selection, reaction time variability and accuracy in tests capturing tonic/phasic alertness and inhibition ranked numerically higher than mean reaction times. While a previous study suggested that objective neuropsychological measures considerably underscored rating scales in distinguishing ADHD from healthy participants^8^, our results show that these scores in general indeed contribute to classification when identifying individuals with ADHD in a mixed help-seeking population. This supports the notion that objective measures like those employed in the current context are indeed important elements of the diagnostic process of ADHD as has been suggested previously^42^.

This study has the following limitations. First, we could not include broader clinical measures such as the CBCL as possible features due to too many missing values. These measures might have provided more specific information on differences between diagnostic entities. Similarly, father ratings also needed to be excluded due to missing data (although father ratings were included in the consistency index where possible). Retraining the classifier without missing data achieved a further increase in the classification accuracy (imputation 66.1% vs no missing data 68.8%). This suggests, that an effort to simplify the diagnostic process in order to reduce the probability of missing data might increase the performance of automated classifiers. Second, although we tested generalizability indirectly using the permutation test, an independent validation sample would provide more precise information on the generalizability of our classifier. Third, the relative importance of single features needs to be interpreted carefully while considering the low classification accuracy differences between the features and the relatively high standard deviations of the achieved accuracies. As the Fig. 1 shows, the standard deviations of the classification accuracies for single features overlapped. Although our results suggest that some features might be superior to others, we cannot conclude that there are single outstanding features in our sample that distinguish individuals with a definite ADHD diagnosis from those with another or no psychiatric diagnosis. Overall, a further increase in classification performance might be achieved by using larger samples with more complete data on all clinically relevant features rather than adding new ones. Our results do not provide full implications for exclusion and/or prioritization of specific clinical ratings in future studies.

## Conclusion

In conclusion, we provide a proof-of-concept that real-world clinical data from medical records might contribute to identification of ADHD among help-seeking individuals. In this context, age, gender, and accuracy/reaction time variability seem to play a marginally more critical role than other features. Further, ADHD core symptoms reported by parents and/or teachers do not seem to carry the degree of importance as it may be assumed. Instead, results suggest a relatively broad combination of symptoms across different domains to indicate an eventual ADHD diagnosis. Overall, this implies that research endeavors aiming to identify biological and less subjective markers of ADHD need to be continued (see Faraone et al.^3^). Although the classification performed above chance (i.e. accuracy of 66.1%), the performance did not reach a level suggestive of possible clinical utility (i.e. 80% accuracy^43^). Multimodal data (particularly neuroimaging and genetic data) might improve the recognition of psychiatric disorders using machine learning^44^. In order to validate such recognition tools, multicentric data are necessary. In order to arrive at firm conclusions in this matter, there is a need for standardized recommendations for ADHD diagnostic procedures, such as specification of the obligatory attention domains, cognitive assessments and assessment scales used. These recommendations should also take into account the risk of missing data finding compromises between a broad assessment and feasibility.

## Supplementary Information

Supplementary Information.
