# Supplementary material for: Training a machine learning classifier to identify ADHD based on real-world clinical data from medical records
Source: Sci Rep. 2022 Jul 28;12:12934. doi: 10.1038/s41598-022-17126-x (PMC9334289; doi:10.1038/s41598-022-17126-x)
Supplement: Supplementary file 1 — Supplementary Information. [file 41598_2022_17126_MOESM1_ESM.pdf]

Training a machine learning classifier to identify ADHD based on real-world clinical data from medical records

Pavol Mikolas, Amirali Vahid, Fabio Bernardoni, Mathilde Süß, Julia Martini, Christian Beste, Annet Bluschke

Supplementary materials

Supplementary note 1: Computation of consistency indices.

Supplementary note 2: The crossvalidation procedure.

Supplementary figure 1: 10-fold crossvalidation procedure.

Supplementary table 1: List of proposed clinical variables and ratings used as features for machine learning classification.

Supplementary table 2: Automated feature selection procedure using the automated, data-driven sequential floating forward selection (SFFS).

Supplementary note 1: Computation of consistency indices.

We computed the consistency indices using the following procedure. We first computed the ratio between the parent and teacher rating for each item. Depending on whether the Conners-3 ratings were available for both parents, or just the mother or just the father, we computed the ratio as R = ((mother + father) / 2) / teacher or R = mother / teacher or R = father / teacher, respectively. Subsequently, we defined a consistency C as: C = exp(-5*abs(log(R))^2). Importantly, abs(log(R)) assesses the distance of R from the perfect consistency point. Indeed, if there is perfect consistency between parents and teacher, then R=1 and abs(log(R))=0). Furthermore, since log(R) = -log(1/R), discrepancies in judgement between parents and teacher are treated symmetrically, and abs(log(R)) is large if either R or 1/R are large. Finally, the exponential function (exp(-5x^2)) is chosen to map high distances into low consistencies and takes values between 0 (no consistency) and 1 (total agreement).

Supplementary note 2: The crossvalidation procedure.

Briefly, we randomly divided the data into ten subsets. We used one subset for testing on each run, whereas the rest of the sample ($k - 1$) was used for training. We repeated the procedure k-times so that all subjects appeared in both testing and training sets. We report the classification accuracies as averages across folds. Where appropriate, we tested the statistical significance of the obtained classification accuracy using a permutation test: Briefly, we randomly assigned the ADHD / non-ADHD labels to all subjects 1000 times and used SVM to predict the group membership of these randomly assigned groups. We calculated the p-value of the accuracy resulting null-hypothesis distribution, i.e., as the proportion of the permutations that yielded a greater accuracy than the accuracy found for the classification model [42,43].

Supplementary figure 1: 10-fold crossvalidation procedure. We randomly divided the data into 10 subsets. On each fold, we assigned one of the 10 subsets to the test set (black), while assigning the rest of the sample to the training set (white). We repeated the procedure 10 times, so that all data were assigned to the training set once. We calculated the classification accuracy as the average accuracy on all folds. Figure adapted from Iniesta et al.[36].

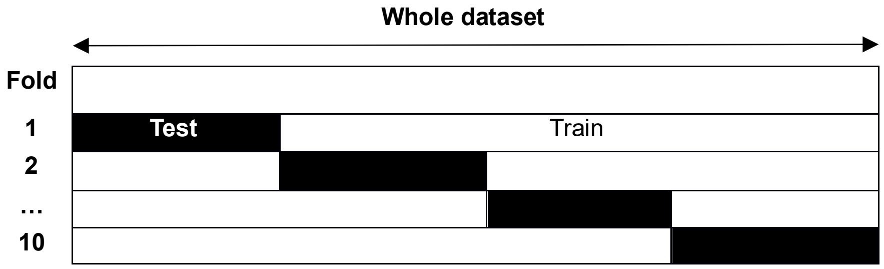

Supplemetary table 1: List of proposed clinical variables and ratings used as features for machine learning classification. p = parent rating (stands both for mother and father ratings), t = teacher rating, r = parent/teacher ratio, RT = reaction time, RT_SD = standard deviation of reaction time.

Domains	Tool of Assessment	Subscales used as features		
**Demographics**		age<br>gender		
**Symptom ratings**	***Conners-3 parent/teacher ratings***	inattention		p/t
		hyperactivity/impulsivity		p/t
		learning problems		p
		executive functions		p
		cognitive problems		t
		aggression		p/t
		peer relations		p/t
		positive impression		p/t
		negative impression		p/t
	Consistency index	inattention		r
		hyperactivity/impulsivity		r
		cognitive problems		r
		aggression		r
		peer relations		r
	Child Behavior Checklist (CBCL) and school equivalent Teacher's Report Form (TRF)	aggressive behavior		p/t
		anxious/depressed		p/t
		attention problems		p/t
		rule-breaking behavior		p/t
		somatic complaints		p/t
		social problems		p/t
		thought problems		p/t
		withdrawn/depressed		p/t
	Strenghts and Difficulties Questionnaire parents (SDQ-E) and teachers (SDQ-L)	emotional symptoms		p/t
		conduct problems		p/t
		hyperactivity/inattention		p/t
		peer relationships problems		p/t
		prosocial behavior		p/t
**Neuropsychology**	***Test battery of attention for adolescents and adults (TAP)***	alertness	tonic<br>phasic	reaction time
				standard devition
		divided attention	auditory<br>visual	reaction time
				standard deviation
				omission errors
		go/ nogo		reaction time
				standard deviation
				omission errors
				commission errors (false alarms)
	Wechsler Intelligence Scale for Children IV-V (WISC IV/V)	general IQ<br>working memory<br>perceptual reasoning<br>verbal comprehension<br>processing speed		

Supplementary table 2: Automated feature selection procedure using the automated, data-driven sequential floating forward selection (SFFS). Each line represents the accuracy achieved during each step of the automated feature selection procedure using that feature along with all previous features (the lines above). The maximum classification accuracy was achieved using a set of 19 features. Using more than 19 features did not improve the accuracy further.

Number of features used	Accuracy	Feature
1	0.576	Gender
2	0.576	Conners_negative impression_t
3	0.579	Go/NoGo_ommission errors
4	0.576	Age
5	0.576	Conners_aggression_t
6	0.599	Conners_peer relations_m
7	0.637	Conners_hyperactivity/impulsivity_t
8	0.647	Conners_inattention_m
9	0.630	WISC_General IQ
10	0.654	Perceptual reasoning
11	0.664	Go/NoGo_reaction time
12	0.613	TAP_Alertness_tonic_reaction time_reaction time
13	0.674	Go/NoGo_commission errors
14	0.671	Verbal comprehension
15	0.671	Conners_hyperactivity/impulsivity_m
16	0.664	TAP_Alertness_Tonic_reaction time_standard deviation
17	0.675	Conners_aggression_m
18	0.671	TAP_Alertness_Phasic_reaction time
19	0.681	TAP_Alertness_Phasic_reaction time_standard deviation
20	0.668	Working memory
